# Oral intake of a combination of glucosyl hesperidin and caffeine elicits an anti-obesity effect in healthy, moderately obese subjects: a randomized double-blind placebo-controlled trial

**DOI:** 10.1186/s12937-016-0123-7

**Published:** 2016-01-19

**Authors:** Tatsuya Ohara, Koutarou Muroyama, Yoshihiro Yamamoto, Shinji Murosaki

**Affiliations:** Research & Development Institute, House Wellness Foods Corporation, 3-20 Imoji, Itami, Hyogo 664-0011 Japan

**Keywords:** Glucosyl hesperidin, Caffeine, Obesity, Body fat, Body mass index

## Abstract

**Background:**

We have previously shown that a combination of glucosyl hesperidin (G-hesperidin) plus caffeine reduces accumulation of body fat, whereas G-hesperidin or caffeine alone shows little effect on high-fat diet-induced obesity in mice. The aim of this study is to evaluate the anti-obesity effect of G-hesperidin plus caffeine on body fat and serum TG in healthy subjects with moderately high body mass index (BMI) and serum TG. Since we considered that there are individual differences in caffeine sensitivity, we conducted dose-finding study of caffeine combined with G-hesperidin.

**Methods:**

Seventy-five healthy subjects with moderately high BMI (24–30 kg/m^2^) and serum TG (100–250 mg/dl) were divided and assigned to 12-week intervention with daily intakes of 500 mg of G-hesperidin with or without 25, 50, or 75 mg of caffeine, or placebo in a randomized double-blind placebo-controlled design .

**Results:**

After intervention, decreases in abdominal fat area (AFA), especially subcutaneous fat area (SFA), were significantly greater in the G-hesperidin with 50-mg caffeine group (AFA:-8.4 ± 21.9 v.s. 16.3 ± 34.1 cm^2^; *p* < 0.05, SFA: -9.3 ± 17.1 v.s. 11.2 ± 18.3 cm^2^; *p* < 0.01) and in the G-hesperidin with 75-mg caffeine group (AFA:-17.0 ± 31.4 v.s. 16.3 ± 34.1 cm^2^; *p* < 0.01, SFA: -12.4 ± 18.7 v.s. 11.2 ± 18.3 cm^2^; *p* < 0.01) than in the placebo group. Fat-decreasing effects of G-hesperidin were enhanced dose-dependently by caffeine addition. BMI decreases were significantly greater in the G-hesperidin with 75-mg caffeine group than in the placebo group (-0.56 ± 0.74 v.s. -0.02 ± 0.58 kg/m^2^; *p* < 0.05). G-hesperidin with/without caffeine had no effect on serum TG (*p* > 0.05 v.s. placebo).

**Conclusions:**

These data suggested that a combination of 500-mg G-hesperidin with 50- or 75-mg caffeine may be useful for the prevention or treatment of obesity.

**Trial registration:**

UMIN Clinical Trials Registry 000019241.

## Background

Prevalence of obesity continues to increase worldwide, mainly as a result of changing lifestyles. It is known that visceral fat-type obesity induces type-2 diabetes mellitus, hyperlipidaemia, hypertension and increases the risk of cardiovascular disease [1–3]. Subcutaneous fat-type obesity is associated with excessive weight gain, and is thought to be the main risk factor for osteoarthritis of the knee [4–7], sleep apnoea [8], and menstrual abnormalities [9–11]. Consequently, research on the prevention and treatment of obesity has been expanded.

Glucosyl hesperidin (G-hesperidin) is synthesized by enzymatic means from hesperidin purified from oranges (*Citrus aurantium*) and dextrin. Its solubility in water is over 10,000-fold greater than that of hesperidin [12]. G-hesperidin has been reported to reduce serum levels of triglyceride (TG) in animals [13–15] and subjects with hypertriglyceridemia [16, 17]. The mechanism of the TG-lowering effect of G-hesperidin is thought to be down-regulation of the synthesis/secretion of very-low-density lipoprotein in hepatocytes [18], inhibition of lipogenesis, and induction of beta oxidation of fatty acids in high-fat diet-fed rats [15]. Studies have shown that daily intake of G-hesperidin (500 mg) in subjects with a moderately high BMI for 12 weeks decreases abdominal fat significantly during the period, but these decreases do not differ significantly from those of subjects taking a placebo [19]. Therefore, the anti-obesity effect of G-hesperidin is incompletely understood.

Caffeine is one of the most widely consumed dietary components (e.g., tea, coffee) with pharmacological effects and psychostimulant activity. Caffeine has been reported to induce lipolysis of adipocytes [20], fat oxidation [21], energy expenditure [22] and thermogenic responses [23] in humans. However, caffeine has not been shown to reduce body fat in humans, so its effects upon fat metabolism are, in general, considered to be insignificant [24].

We have shown before that combining food ingredients involved in lipid metabolism is useful for the prevention or treatment of obesity. For example, a mixture of thiamine, L-arginine, caffeine, and citric acid has been shown to have an anti-obesity effect in obese mice and humans with a high BMI [25, 26]. We have also shown that a combination of G-hesperidin and caffeine reduces accumulation of body fat through (at least in part) inhibition of hepatic lipogenesis, whereas G-hesperidin or caffeine alone show little effect on high-fat diet-induced obesity in mice [27].

We conducted an exploratory trial to investigate the anti-obesity effects of a combination of G-hesperidin and caffeine in subjects with a BMI of 24–30 kg/m^2^ and serum levels of TG of 100–250 mg/dl at a potentially effective dose of G-hesperidin (500 mg) [19] with that of caffeine (25, 50, or 75 mg) [26].

## Methods

### Test samples

Composition of test samples is listed in Table 1. The base tablet comprised lactose, cornstarch, hard starch, and sugar ester. Lactose was substituted by G-hesperidin and caffeine. G-hesperidin was provided from Hayashibara (Okayama, Japan) and contained monoglucosyl hesperidin (>75.0 %) and non-glycosylated hesperidin (<25.0 %), which was confirmed by high-performance liquid chromatography. Caffeine (purity, >98.5 %), extracted from coffee beans, was purchased from Shiratori Pharmaceuticals (Chiba, Japan).

**Table 1:** Composition of test samples (3 tablets/1 g)

	Placebo	GH	GH + Caf 25	GH + Caf 50	GH + Caf 75
Energy (kcal)	4.05	4.01	4.01	4.01	4.01
Protein (g)	0	0	0	0	0
Fat (g)	0.03	0.03	0.03	0.03	0.03
Carbohydrate (g)	0.94	0.93	0.93	0.93	0.93
Glucosyl hesperidin (mg)	0	500	500	500	500
Caffeine (mg)	0	0	25	50	75

*GH* glucosyl hesperidin 500 mg, *Caf 25, 50, 75* caffeine 25 mg, 50 mg, 75 mg

### Subjects

A total of 160 Japanese people subjects were evaluated for eligibility. Screening led to a study cohort of 38 men and 37 women (20–65 years) with a moderately high BMI (24–30 kg/m^2^) and serum level of TG (100–250 mg/dL). Exclusion criteria were patients who had (i) ingested medicines and/or health-foods that might affect obesity and serum levels of TG or (ii) consumed many beverages containing caffeine (e.g. tea, green tea, coffee, and cola). From the results of a pre-questionnaire, daily caffeine consumption of each group was estimated as G-hesperidin, 112 ± 104 mg/day; G-hesperidin with 25-mg caffeine, 111 ± 76 mg/day; G-hesperidin with 50-mg caffeine, 89 ± 86 mg/day; G-hesperidin with 75-mg caffeine, 145 ± 104 mg/day; and placebo, 112 ± 84 mg/day.

### Trial design

This trial was carried out from April to August 2013 on subjects at Fukuhara Clinic (Hokkaido, Japan) and complied with the Declaration of Helsinki. The study protocol was approved by the Ethics Review Board of Miyawaki Orthopedic Clinic (Hokkaido, Japan). Procedures were explained fully to subjects. Written informed consent was obtained from each subject before study commencement.

This was a randomised, double-blind placebo-controlled trial comprising periods of pre-checking (1 week), eligibility assessment (4 weeks), intervention (12 weeks), and post-ingestion (4 weeks).

**Fig. 1:**
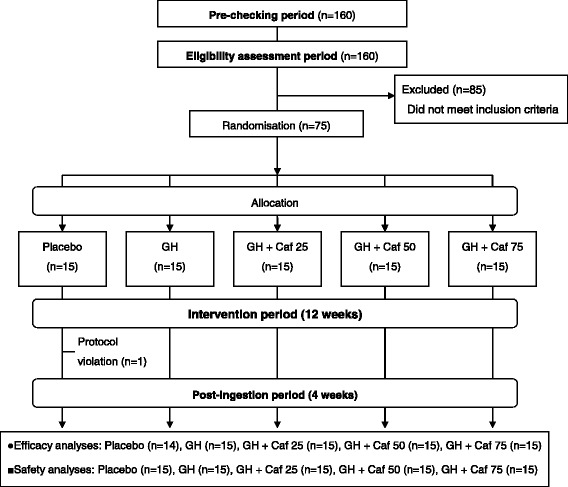
Study flowchart. *GH* glucosyl hesperidin 500 mg, *Caf 25, 50, 75* caffeine 25 mg, 50 mg, 75 mg

After assessment of eligibility, 75 subjects were allocated into five groups: 500-mg G-hesperidin; 500-mg G-hesperidin with 25-mg caffeine; 500-mg G-hesperidin with 50-mg caffeine; 500-mg G-hesperidin with 75-mg caffeine; placebo (Fig. 1). Allocation was by stratified randomisation based on age, sex, and waist circumference.

### Intervention

Subjects ingested test samples as tablets (Table 1) every day for 12 weeks. Time of ingestion of the test sample was not dictated to patients, but was recommended to be in the morning. Ingestion of large quantities of caffeine was prohibited. Ingestion of health-foods that influence levels of TG or cholesterol, or that affect obesity, was prohibited. Use of drugs for prolonged periods and use of drugs that interact with caffeine was banned. However, if a subject had been instructed to use a drug by his/her physician, he/she reported its name, dose, period, and indication. Dieting, drinking excessive amounts of alcohol, and intake of medicines known to affect lipid metabolism was prohibited. Subjects were instructed to maintain their regular lifestyle, in particular in relation to meal size and amount of exercise.

After assessment of eligibility, subjects visited the clinic five times at 4-week intervals. Subjects were prohibited from eating and drinking except for water from 9 pm the day before their visit until all checkups had been completed.

At the day of the visit, subjects underwent a physical examination, and analyses of blood and urine were conducted. The diet record, walking record, and diary were submitted at this time. At week 0 and week 12, the abdominal fat area (AFA) was measured.

### Measurement of AFA

Umbilical AFA was measured using a computed tomography system (CT-W450; Hitachi Medical Corporation, Tokyo, Japan) in accordance with the method described by Tokunaga et al. [28]. Total fat area (TFA), visceral fat area (VFA), and subcutaneous fat area (SFA) were computed using Fat Scan™ v3.0 (N2 systems, Osaka, Japan).

### Safety assessment

Haematological assessments (counts of white blood cells, red blood cells and platelets, haemoglobin, haematocrit, mean corpuscular volume, mean corpuscular haemoglobin, mean corpuscular haemoglobin concentration), blood biochemical assessment (total protein, albumin, total bilirubin, aspartate aminotransferase, alanine transaminase, lactate dehydrogenase, alkaline phosphatase, gamma-glutamyl transferase, total cholesterol, high-density lipoprotein-cholesterol (HDL-C), low-density lipoprotein-cholesterol (LDL-C), TG, glucose, uric acid, urea nitrogen, creatinine, Na^+^, K^+^, Cl^–^, Hb_A1c_), and urine analyses (specific gravity, pH, protein, sugar, urobilinogen, bilirubin, ketone bodies, occult blood reaction) were conducted at Daiichi-Kishimoto Clinical Laboratory (Sapporo, Japan). Anthropometric measurements were undertaken at Fukuhara Clinic.

### Diet and walking surveys

During the intervention period and post-ingestion period, subjects photographed all the meals (including snacks) that they had consumed and recorded their contents in a diet questionnaire for 3 days before the checkup. A nutritionist calculated values of energy, protein, lipid, and carbohydrate using Excel Eiyokun™ v6.0 (Kenpakusha, Tokyo, Japan) from the diet questionnaires and photographs. During the same 3 days as diet recording, the number of steps was documented (using a pedometer) as the exercise volume.

### Subject diaries

During the intervention period and post-ingestion period, subjects recorded the time of ingestion of the test sample and subjective symptoms in their diaries.

### Statistical analyses

Values are the mean ± SD. Comparison between baseline (week 0) and each time-point in each group was done by the paired *t*-test with multiple corrections except for AFA. After checking the equality of variance by the Bartlett method, comparisons between groups were analysed by Dunnett’s method in the case of equal variance, and by Steel’s method in the case of unequal variance. Analyses were performed using SAS v9.3 (SAS Institute, Cary, NC, USA), and *p* < 0.05 was considered significant.

## Results

### Baseline characteristics of study participants

Seventy-five subjects completed the study. However, one subject who completed the study with a major violation of the test protocol owing to bereavement was excluded from efficacy analyses. The baseline (week 0) characteristics of 74 subjects are listed in Table 2. No significant difference was observed in baseline (week 0) characteristics between groups. Safety analyses were conducted in all 75 subjects. The study flowchart is shown in Fig. 1.

**Table 2:** Subjects’ baseline characteristics

	Placebo	GH	GH + Caf 25	GH + Caf 50	GH + Caf 75
Subject (Men/women)	N = 14 (7/7)	n = 15 (7/8)	n = 15 (8/7)	n = 15 (8/7)	n = 15 (7/8)
Age (y)	49.4 ± 8.5	49.0 ± 10	49.1 ± 13	50.8 ± 9.8	47.3 ± 9.9
Height (cm)	163.7 ± 11	162.9 ± 9.7	162.0 ± 8.9	162.6 ± 8.4	162.6 ± 7.3
Body weight (g)	70.3 ± 9.6	70.0 ± 9.9	69.2 ± 9.0	70.5 ± 7.4	70.2 ± 6.5
BMI (kg/m^2^)	26.1 ± 1.1	26.2 ± 1.6	26.3 ± 1.2	26.7 ± 1.8	26.5 ± 1.2
Waist (cm)	90.9 ± 5.7	90.6 ± 3.0	90.4 ± 5.3	90.0 ± 4.7	91.0 ± 4.4
Hip (cm)	96.5 ± 5.1	96.8 ± 2.9	95.9 ± 4.4	97.1 ± 3.1	96.4 ± 4.6
Abdominal fat area					
Total (cm^2^)	301.7 ± 50.6	304.3 ± 41.8	297.9 ± 54.8	295.1 ± 53.7	310.8 ± 43.9
Visceral (cm^2^)	97.2 ± 53.1	97.6 ± 27.7	107.5 ± 42.3	108.1 ± 46.1	101.1 ± 28.9
Subcutaneous (cm^2^)	204.6 ± 59.5	206.6 ± 45.4	190.4 ± 59.0	187.0 ± 48.0	209.8 ± 45.4

*GH* glucosyl hesperidin 500 mg, *Caf 25, 50, 75* caffeine 25 mg, 50 mg, 75 mg, *BMI* Body Mass Index

Mean ± SD

### Ingestion of test samples

Percentage of patients who ingested test samples in the placebo group, 500-mg G-hesperidin group, 500-mg G-hesperidin with 25-mg caffeine group, 500-mg G-hesperidin with 50-mg caffeine group, and 500-mg G-hesperidin with 75-mg caffeine group was 99.4, 99.6, 99.8, 99.8, and 99.8 %, respectively.

### Energy intake and exercise volume

Energy intake and exercise volume throughout the intervention period and post-ingestion period are shown in Table 3. Occasionally, significant differences were found within groups, but there was no significant difference between groups.

**Table 3:** Dietary composition and exercise volume during intervention period and post- ingestion period

			Intervention period				Post-ingestion period
Item	Group	n	Week-0	Week-4	Week-8	Week-12	Week-16
Energy (kcal/3d)	Placebo	14	6075 ± 1336	5925 ± 934	5648 ± 1108**	5623 ± 872	5488 ± 924*
	GH	15	5599 ± 1387	5748 ± 1500	5383 ± 1250	5322 ± 1259	5328 ± 1013
	GH + Caf 25	15	5606 ± 1270	5512 ± 1031	5252 ± 1340	4981 ± 1291	5155 ± 1395
	GH + Caf 50	15	5240 ± 1463	5774 ± 1666	5425 ± 1538	5302 ± 1723	5272 ± 1635
	GH + Caf 75	15	5727 ± 929	5268 ± 1369	5546 ± 1302	5119 ± 1538	5401 ± 1618
Exercise volume (step/3d)	Placebo	14	21661 ± 12162	22372 ± 9366	27058 ± 11735	25258 ± 10247	23994 ± 10039
	GH	15	17118 ± 7809	22161 ± 15033	19231 ± 8677	22738 ± 11305*	20298 ± 8622
	GH + Caf 25	15	19929 ± 8679	22946 ± 14412	22350 ± 16172	19656 ± 16855	22996 ± 14817
	GH + Caf 50	15	23513 ± 13156	21881 ± 12157	22491 ± 9911	23005 ± 12524	24650 ± 12288
	GH + Caf 75	15	18843 ± 7455	19828 ± 6532	24016 ± 8703	24036 ± 9488	23972 ± 8216

*GH* glucosyl hesperidin 500 mg, *Caf 25, 50, 75* caffeine 25 mg, 50 mg, 75 mg

Data are the mean ± SD recorded 3 days before each check-up

*p < 0.05, **p < 0.01 vs. baseline (week-0), (paired t-test)

### Effect of a combination of G-hesperidin and caffeine upon obesity

**Fig. 2:**
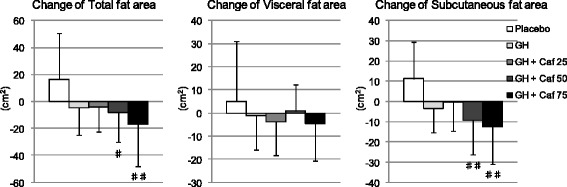
Change in abdominal fat area after 12 weeks of intervention. Data are expressed as Mean ± SD. ♯:*p* < 0.05, ♯♯:*p* < 0.01 v.s. Placebo. *n* = 15 in each of groups except for Placebo (*n* = 14). *GH* glucosyl hesperidin 500 mg, *Caf 25, 50, 75* caffeine 25 mg, 50 mg, 75 mg

Measurement at week 0 and week 12 as well as change from the baseline (week 0) in AFA is shown in Table 4. Furthermore, changes from baseline are also represented in Fig. 2. Decreases in TFA in the G-hesperidin with 50-mg caffeine group and in the G-hesperidin with 75-mg caffeine group were significantly greater than those in the placebo group. Degree of decrease of TFA appeared to be enhanced in a caffeine dose-dependent manner. Decreases in SFA in the G-hesperidin with 50-mg caffeine group and G-hesperidin with 75-mg caffeine group were significantly greater than those in the placebo group. However, there were no significant decreases in VFA between the placebo group and groups that ingested G-hesperidin with or without caffeine.

**Table 4:** Effect of G-hesperidin + caffeine tablets on abdominal fat area

Parameter	Group	n	Week-0	Week-12	Change from baseline
Total fat area (cm^2^)	Placebo	14	301.7 ± 50.6	318.1 ± 50.4	16.3 ± 34.1
	GH	15	304.3 ± 41.8	299.7 ± 44.1	−4.6 ± 20.5
	GH + Caf 25	15	297.9 ± 54.8	293.7 ± 61.5	−4.2 ± 18.2
	GH + Caf 50	15	295.1 ± 53.7	286.7 ± 48.9	−8.4 ± 21.9^#^
	GH + Caf 75	15	310.8 ± 43.9	293.9 ± 45.5	−17.0 ± 31.4^##^
Visceral fat area (cm^2^)	Placebo	14	97.2 ± 53.1	102.3 ± 61.9	5.1 ± 25.9
	GH	15	97.6 ± 27.7	96.6 ± 27.5	−1.1 ± 15.0
	GH + Caf 25	15	107.5 ± 42.3	103.6 ± 40.0	−3.8 ± 14.5
	GH + Caf 50	15	108.1 ± 46.1	109.0 ± 46.6	0.9 ± 11.3
	GH + Caf 75	15	101.1 ± 28.9	96.5 ± 33.7	−4.5 ± 16.1
Subcutaneous fat area (cm^2^)	Placebo	14	204.6 ± 59.5	215.8 ± 63.5*	11.2 ± 18.3
	GH	15	206.6 ± 45.4	203.1 ± 44.2	−3.5 ± 11.8
	GH + Caf 25	15	190.4 ± 59.0	190.1 ± 60.1	−0.4 ± 14.3
	GH + Caf 50	15	187.0 ± 48.0	177.7 ± 44.0	−9.3 ± 17.1^##^
	GH + Caf 75	15	209.8 ± 45.4	197.3 ± 44.4*	−12.4 ± 18.7^##^

*GH* glucosyl hesperidin 500 mg, *Caf 25, 50, 75* caffeine 25 mg, 50 mg, 75 mg

Mean ± SD, *p < 0.05 *vs*. baseline (week-0), (paired *t*-test), ^#^p < 0.05, ^##^p < 0.01 *vs*. placebo

Measurements during and after the intervention period as well as changes from baseline (week 0) in body weight, the BMI, and circumferences of the waist and hip are shown in Table 5. Decreases in body weight at week 8 (*p* = 0.045) but not at week 12 (*p* = 0.089) were significantly greater in the G-hesperidin with 75-mg caffeine group than in the placebo group. Decreases in the BMI in the G-hesperidin with 75-mg caffeine group were significantly greater than those in the placebo group at week 8 and week 12. The BMI-decreasing effect of G-hesperidin appeared to be enhanced dose-dependently by caffeine addition. Decreases in waist circumference were significantly greater in the G-hesperidin with 25 mg caffeine group than in the placebo group at week-8 and at week-12. Similar decreases were observed in the G-hesperidin with 75 mg caffeine group but were not significant. There were no significant differences in decreases in hip circumference between the placebo group, and groups that ingested G-hesperidin with or without caffeine.

**Table 5:** Anthropometric variables during the intervention period and post- ingestion periods

			Intervention period				post- ingestion period
Parameter	Group	n	Week-0	Week-4	Week-8	Week-12	Week-16
Body weight (kg)	Placebo	14	70.3 ± 9.6	70.1 ± 9.2	70.6 ± 9.0	70.1 ± 8.8	70.5 ± 9.3
	GH	15	70.0 ± 9.9	69.5 ± 9.7	69.9 ± 9.7	69.6 ± 9.4	70.0 ± 9.7
	GH + Caf 25	15	69.2 ± 9.0	69.1 ± 9.0	68.8 ± 9.2	68.6 ± 9.5	68.7 ± 9.5
	GH + Caf 50	15	70.5 ± 7.4	70.2 ± 7.5	70.1 ± 7.7	69.8 ± 7.7	69.8 ± 8.0
	GH + Caf 75	15	70.2 ± 6.5	69.8 ± 6.6	69.3 ± 6.9	68.8 ± 7.0	68.9 ± 7.0
ΔBody weight (kg)	Placebo	14		−0.26 ± 1.1	0.24 ± 1.2	−0.19 ± 1.7	0.16 ± 1.7
	GH	15		−0.44 ± 1.0	−0.09 ± 0.9	−0.39 ± 1.1	0.08 ± 1.4
	GH + Caf 25	15		−0.11 ± 0.8	−0.39 ± 1.0	−0.57 ± 1.1	−0.48 ± 1.7
	GH + Caf 50	15		−0.29 ± 1.1	−0.44 ± 1.3	−0.67 ± 1.4	−0.67 ± 1.8
	GH + Caf 75	15		−0.36 ± 1.0	−0.88 ± 1.4^#^	−1.43 ± 2.0	−1.27 ± 2.2
BMI (kg/m^2^)	Placebo	14	26.1 ± 1.1	26.1 ± 1.2	26.3 ± 1.1	26.1 ± 1.3	26.2 ± 1.2
	GH	15	26.2 ± 1.6	26.1 ± 1.6	26.2 ± 1.6	26.1 ± 1.6	26.3 ± 1.6
	GH + Caf 25	15	26.3 ± 1.2	26.2 ± 1.3	26.1 ± 1.3	26.0 ± 1.4	26.1 ± 1.6
	GH + Caf 50	15	26.7 ± 1.8	26.6 ± 1.8	26.5 ± 1.9	26.4 ± 1.9	26.4 ± 2.0
	GH + Caf 75	15	26.5 ± 1.2	26.4 ± 1.3	26.2 ± 1.3	25.9 ± 1.3*	26.0 ± 1.3
ΔBMI (kg/m^2^)	Placebo	14		−0.07 ± 0.37	0.14 ± 0.39	−0.02 ± 0.58	0.08 ± 0.57
	GH	15		−0.15 ± 0.36	−0.01 ± 0.38	−0.13 ± 0.42	0.03 ± 0.53
	GH + Caf 25	15		−0.05 ± 0.31	−0.14 ± 0.40	−0.23 ± 0.40	−0.19 ± 0.65
	GH + Caf 50	15		−0.13 ± 0.42	−0.19 ± 0.52	−0.28 ± 0.56	−0.27 ± 0.70
	GH + Caf 75	15		−0.13 ± 0.41	−0.35 ± 0.55^#^	−0.56 ± 0.74^#^	−0.51 ± 0.84
Waist (cm)	Placebo	14	90.9 ± 5.7	90.7 ± 5.5	91.1 ± 5.1	91.3 ± 4.8	91.3 ± 5.3
	GH	15	90.6 ± 3.0	90.6 ± 3.5	90.6 ± 3.4	90.6 ± 3.3	90.6 ± 3.8
	GH + Caf 25	15	90.4 ± 5.3	89.9 ± 5.9	89.3 ± 6.0*	89.1 ± 6.2*	89.5 ± 6.4
	GH + Caf 50	15	90.0 ± 4.7	89.6 ± 5.0	89.7 ± 5.2	89.1 ± 5.8	88.8 ± 6.5
	GH + Caf 75	15	91.0 ± 4.4	91.0 ± 3.9	90.3 ± 4.0	89.9 ± 3.5	89.8 ± 3.1
ΔWaist (cm)	Placebo	14		−0.12 ± 0.9	0.19 ± 1.4	0.44 ± 2	0.42 ± 2.1
	GH	15		0.01 ± 1.0	−0.01 ± 1.4	−0.02 ± 1.3	−0.03 ± 1.3
	GH + Caf 25	15		−0.49 ± 1.1	−1.17 ± 1.4^#^	−1.31 ± 1.6^#^	−0.96 ± 2.2
	GH + Caf 50	15		−0.31 ± 1.1	−0.28 ± 1.3	−0.82 ± 1.9	−1.12 ± 2.6
	GH + Caf 75	15		0.05 ± 1.0	−0.66 ± 0.9	−1.11 ± 1.7	−1.21 ± 2.2
Hip (cm)	Placebo	14	96.5 ± 5.1	96.3 ± 5.3	96.5 ± 5.1	96.9 ± 5.5	97.1 ± 5.6
	GH	15	96.8 ± 2.9	96.6 ± 2.8	96.9 ± 2.8	96.4 ± 3.1	96.6 ± 2.8
	GH + Caf 25	15	95.9 ± 4.4	96.1 ± 4.0	96.1 ± 4.0	96.0 ± 4.5	96.0 ± 5.0
	GH + Caf 50	15	97.1 ± 3.1	96.8 ± 3.2	97.0 ± 3.2	97.0 ± 3.5	97.0 ± 3.4
	GH + Caf 75	15	96.4 ± 4.6	96.2 ± 4.6	96.0 ± 4.6	95.5 ± 4.5	95.6 ± 4.6
ΔHip (cm)	Placebo	14		−0.17 ± 0.7	0.01 ± 1.1	0.37 ± 1.6	0.64 ± 1.4
	GH	15		−0.17 ± 0.8	0.05 ± 1.3	−0.41 ± 1.2	−0.23 ± 1.5
	GH + Caf 25	15		0.23 ± 0.8	0.17 ± 1.0	0.13 ± 1.4	0.06 ± 2.0
	GH + Caf 50	15		−0.26 ± 0.8	−0.11 ± 1.3	−0.08 ± 1.7	−0.13 ± 2.0
	GH + Caf 75	15		−0.21 ± 1.0	−0.39 ± 1.2	−0.85 ± 1.8	−0.79 ± 2.0

*GH* glucosyl hesperidin 500 mg, *Caf 25, 50, 75* caffeine 25 mg, 50 mg, 75 mg, *BMI* Body Mass Index

Mean ± SD, *p < 0.05, *vs*. baseline (week-0), (paired *t*-test), ^#^p < 0.05 *vs*. placebo (Dunnett’s test or Steel’s test)

### Effect of a combination of G-hesperidin and caffeine on serum levels of lipids

Measurements during and after the intervention period as well as changes from baseline (week 0) in serum levels of TG, total cholesterol, HDL-C and LDL-C are shown in Table 6. G-hesperidin has been reported to lower the serum levels of TG in subjects with moderately high levels of TG in serum [17]. However, TG levels in the G-hesperidin-alone group did not decrease during the intervention period, and the changes from baseline (week 0) were not different from those of the placebo group. TG levels in the G-hesperidin with caffeine group also did not decrease. Occasionally, serum levels of total cholesterol, HDL-C, and LDL-C decreased in G-hesperidin with/without caffeine groups during intervention periods but changes from baseline (week 0) were not different from those in the placebo group.

**Table 6:** Blood biochemical parameters during the intervention period and post- ingestion period

				Intervention period				post- ingestion period
Parameter	Standard value	Group	n	Week-0	Week-4	Week-8	Week-12	Week-16
Triglyceride (mg/dl)	50 ~ 149	Placebo	14	144 ± 65	142 ± 43	131 ± 55	141 ± 61	119 ± 44
		GH	15	128 ± 55	132 ± 49	134 ± 71	153 ± 70	152 ± 58
		GH + Caf 25	15	126 ± 48	137 ± 53	135 ± 47	129 ± 36	127 ± 47
		GH + Caf 50	15	133 ± 70	137 ± 29	136 ± 50	148 ± 66	144 ± 54
		GH + Caf 75	15	156 ± 74	136 ± 35	127 ± 40	134 ± 39	135 ± 41
ΔTriglyceride (mg/dl)		Placebo	14		−1.4 ± 58	−12.4 ± 49	−2.6 ± 57	−24.9 ± 50
		GH	15		4.3 ± 33	5.5 ± 46	24.6 ± 60	24.0 ± 47
		GH + Caf 25	15		11.1 ± 49	8.9 ± 51	3.0 ± 44	1.0 ± 46
		GH + Caf 50	15		4.4 ± 69	2.9 ± 70	15.3 ± 45	10.7 ± 54
		GH + Caf 75	15		−21.9 ± 80	−29.3 ± 66	−22.7 ± 61	−21.3 ± 74
Total cholesterol (mg/dl)	150 ~ 219	Placebo	14	220 ± 37	216 ± 44	215 ± 39	220 ± 39	219 ± 45
		GH	15	229 ± 29	217 ± 29*	224 ± 29	224 ± 30	222 ± 28
		GH + Caf 25	15	213 ± 27	210 ± 24	202 ± 30	205 ± 26	205 ± 31
		GH + Caf 50	15	222 ± 34	218 ± 37	216 ± 34	222 ± 34	219 ± 40
		GH + Caf 75	15	241 ± 27	228 ± 31	225 ± 27*	227 ± 24*	233 ± 23
ΔTotal cholesterol (mg/dl)		Placebo	14		−4.8 ± 20	−5.2 ± 16	−0.1 ± 18	−1.9 ± 24
		GH	15		−12.5 ± 17	−5.3 ± 15	−5.8 ± 14	−7.6 ± 16
		GH + Caf 25	15		−3.3 ± 17	−11.3 ± 21	−8.2 ± 16	−8.5 ± 17
		GH + Caf 50	15		−4.5 ± 16	−6.1 ± 18	0 ± 15	−3.2 ± 22
		GH + Caf 75	15		−5.3 ± 22	−15.5 ± 21	−13.1 ± 14	−7.6 ± 22
HDL-cholesterol (mg/dl)	Men:	Placebo	14	54.6 ± 12	51.8 ± 10	51.5 ± 12	51.8 ± 11	53.0 ± 12
	40 ~ 80	GH	15	53.9 ± 10	51.8 ± 10	52.3 ± 9	50.9 ± 9	51.7 ± 9
	Women:	GH + Caf 25	15	53.9 ± 11	53.4 ± 13	49.3 ± 10**	49.9 ± 11**	50.9 ± 10
	40 ~ 90	GH + Caf 50	15	53.6 ± 11	51.5 ± 9	49.7 ± 6	50.1 ± 8	51.7 ± 8
		GH + Caf 75	15	56.5 ± 11	55.9 ± 12	55.0 ± 12	54.3 ± 11	55.1 ± 10
ΔHDL-cholesterol (mg/dl)		Placebo	14		−2.8 ± 6.2	−3.1 ± 4.7	−2.8 ± 5.0	−1.6 ± 5.6
		GH	15		−2.1 ± 5.4	−1.6 ± 4.4	−3.0 ± 5.6	−2.1 ± 4.8
		GH + Caf 25	15		−0.5 ± 4.9	−4.6 ± 4.4	−4.0 ± 3.9	−3.1 ± 4.4
		GH + Caf 50	15		−2.1 ± 5.1	−3.9 ± 7.1	−3.5 ± 4.8	−1.9 ± 8.2
		GH + Caf 75	15		−0.3 ± 6.7	−1.5 ± 6.4	−2.2 ± 5.7	−1.4 ± 5.8
LDL-cholesterol (mg/dl)	70 ~ 139	Placebo	14	147 ± 41	142 ± 44	145 ± 40	144 ± 39	148 ± 47
		GH	15	151 ± 29	140 ± 24	148 ± 30	140 ± 30*	144 ± 25
		GH + Caf 25	15	140 ± 30	137 ± 20	134 ± 27	133 ± 25	134 ± 27
		GH + Caf 50	15	149 ± 37	147 ± 40	147 ± 36	148 ± 36	147 ± 37
		GH + Caf 75	15	161 ± 28	155 ± 29	152 ± 19	149 ± 21	160 ± 24
ΔLDL-cholesterol (mg/dL)		Placebo	14		−5.6 ± 17	−2.4 ± 15	−2.8 ± 16	0.9 ± 21
		GH	15		−11.0 ± 17	−3.1 ± 15	−10.6 ± 14	−7.5 ± 14
		GH + Caf 25	15		−2.6 ± 21	−5.9 ± 18	−7.5 ± 14	−5.9 ± 15
		GH + Caf 50	15		−2.7 ± 16	−2.3 ± 18	−1.0 ± 14	−2.8 ± 15
		GH + Caf 75	15		1.1 ± 31	−8.9 ± 20	−12.2 ± 21	−1.5 ± 23

GH glucosyl hesperidin 500 mg, Caf 25, 50, 75, caffeine 25 mg, 50 mg, 75 mg, BMI Body Mass Index, LDL low-density lipoprotein, HDL high-density lipoprotein

Mean ± SD *p < 0.05, **P < 0.01 vs. baseline (week 0) (paired t-test)

### Safety assessment

Twenty-nine adverse events (AEs) were recorded during the study. Of the seven AEs in the placebo group, three were symptoms of a common cold, one was a pollen allergy, one was pain, one was tinnitus, and one was sudden deafness. Of the six AEs in the G-hesperidin group, three were symptoms of a common cold, two were pain, and one was conjunctivitis. Of the five AEs in the G-hesperidin with 25 mg caffeine group, two were symptoms of a common cold, two were digestive symptoms, and one was pain. Of the eight AEs in the G-hesperidin with 50-mg caffeine group, three were digestive symptoms, one was a symptom of a common cold, one was pain, one was a pollen allergy, one was fatigue, and one was irritation. Of three AEs in the G-hesperidin with 75-mg caffeine group, one was a symptom of common cold, one was pain, and one was a pollen allergy. Of the 29 AEs, one was moderate and 28 were mild. All AEs were judged to be unrelated to the dietary intervention, in which the moderate AE of sudden deafness was considered to be a result of the stress of bereavement.

Among the safety assessments undertaken, total protein in serum at week 4 in the G-hesperidin with 25 mg caffeine group (7.24 ± 0.23 g/dl) or at week 8 in the G-hesperidin with 75 mg caffeine group (7.27 ± 0.39 g/dl) were significantly greater than in the placebo group (week 4: 6.94 ± 0.19 g/dl, week 8: 7.27 ± 0.39 g/dl), and urine gravity at week 4 in the G-hesperidin with 75 mg caffeine group (1.013 ± 0.004 g/ml) was significantly lower than in the placebo group (1.018 ± 0.006 g/ml). However, these changes were within the ranges of corresponding reference values.

## Discussion

We conducted a randomised double-blind placebo-controlled study to evaluate the anti-obesity effect and serum TG-lowering effect of a combination of G-hesperidin and caffeine in subjects with a moderately high BMI and moderately high serum levels of TG.

Intake of G-hesperidin with 50-mg caffeine and 75-mg caffeine showed significant decreasing effects on AFA (especially on SFA). This fat-decreasing effect seemed to be enhanced by the dose-dependent addition of caffeine. Significant decreases in body weight and the BMI were observed in the G-hesperidin with 75-mg caffeine group compared with those in the placebo group. Decreases in VFA in G-hesperidin with caffeine groups were not significant compared with those in the placebo group. Visceral adipose tissue has been proposed to mediate obesity-related unfavourable levels of insulin, glucose and lipids, but subcutaneous abdominal adipose tissue has been shown to be independent risk factor for the metabolic complications of obesity [29–31]. Therefore, our observations that a combination of G-hesperidin and caffeine decreased AFA may have clinical significance.

**Fig. 3:**
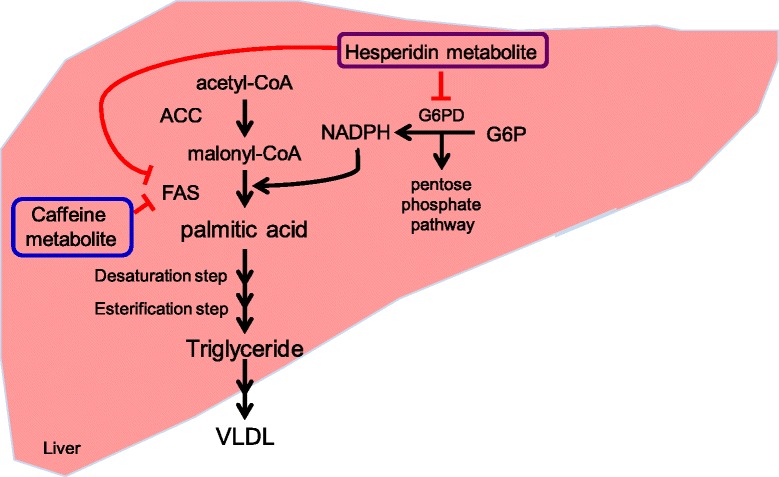
Fat-reducing effect of the combination of G-hesperidin and caffeine is partially through the inhibition of hepatic lipogenesis. G6P, Glucose-6-phosphate; G6PD, Glucose-6-phosphate dehydrogenase; ACC, Acetyl-CoA carboxylase; FAS, Fatty acid synthase; VLDL, very low density lipoprotein

We have developed anti-obesity agents containing dietary components that affect lipid metabolism. Their effectiveness has been evaluated in non-insulin-dependent diabetic KK mice induced to develop obesity by feeding with a high-fat diet. Among the agents examined in this model, a mixture of thiamine, arginine, caffeine, and citric acid, as well as a mixture of arginine, caffeine, soy isoflavones and carnitine, have shown prominent anti-obesity effects [25, 32]. Recently, we have shown that a combination of G-hesperidin and caffeine decreased weights of subcutaneous fat and visceral fat effectively in high-fat diet-induced obese mice [27]. We consider the mechanism of its anti-obesity effect is partially through the inhibition of hepatic lipogenesis (Fig. 3) [27]. In addition to the effectiveness of a mixture of thiamine, arginine, caffeine and citric acid in the treatment of obesity in humans [26], we showed an anti-obesity effect of a combination of G-hesperidin and caffeine in moderately obese subjects in the present study. Therefore, the effectiveness of anti-obesity agents comprising dietary components observed in obese KK mice can be expected to have a similar effect in humans.

Reports have shown that 12-week intake of G-hesperidin (500 mg) lowers serum levels of TG in subjects with moderately high levels of TG in serum [17]. In contrast to those observations, intake of G-hesperidin with or without caffeine had no effect on serum levels of TG in subjects with moderately high levels of TG. Unlike previous studies, we recruited subjects with a moderately high BMI in addition to a moderately high level of TG. It has been reported that plasma levels of TG in obese subjects do not decrease efficiently in response to insulin, are overproduced, or are resistant to clearance [33]. Such changes in TG metabolism in obese subjects may affect the action of G-hesperidin, leading to the slight effect of G-hesperidin on serum levels of TG in obese subjects.

In the present study, mean daily consumption of caffeine other than during intervention was about 100 mg in subjects of each group. Nevertheless, a dose–response of caffeine from 25 mg to 75 mg was observed in the anti-obesity effects of this combination. Hence, concomitant (but not habitual) intake of caffeine with G-hesperidin might be important for the anti-obesity activity of this combination. Indeed, in studies demonstrating the anti-obesity effect of caffeine-containing components, caffeine was taken concomitantly with other ingredients [34, 35]. If concomitant intake is important, then whether single intake of such a low dose of caffeine has physiological relevance is of particular importance. It has been reported that a single dose of 12.5 mg caffeine affects cognitive performance [36] and that 32-mg caffeine improves auditory vigilance and visual reaction time [37]. Therefore, concomitant intake of caffeine may have physiological activity and synergize with G-hesperidin. Further investigations are required to clarify the importance of concomitant intake of caffeine and G-hesperidin.

## Conclusions

Our results showed that intake of a combination of 500-mg G-hesperidin and 75-mg caffeine for 12 weeks significantly reduced abdominal fat (especially subcutaneous fat), body weight and the BMI in subjects with a moderately high BMI. Therefore, a combination of G-hesperidin and caffeine may be useful for the prevention or treatment of obesity.

## Abbreviations

AFA: abdominal fat area
BMI: body mass index
G-hesperidin: glucosyl hesperidin
TG: triglyceride
