# Erratum to: Oral intake of a combination of glucosyl hesperidin and caffeine elicits an antiobesity effect in healthy, moderately obese subjects: a randomized double-blind placebo-controlled trial

**DOI:** 10.1186/s12937-017-0253-6

**Published:** 2017-05-12

**Authors:** Tatsuya Ohara, Koutarou Muroyama, Yoshihiro Yamamoto, Shinji Murosaki

**Affiliations:** Research & Development Institute, House Wellness Foods Corporation, 3-20 Imoji, Itami, Hyogo 664-0011 Japan

## Erratum

After publication of this article [1] it was noticed there were errors in the paper regarding the dose and grade of glucosyl hesperidin due to miscommunications.

The composition of glucosyl hesperidin is reported as “500 mg” but this should instead be listed as “470 mg” throughout the article. Additionally, the G-hesperidin should be reported as containing 74% monoglucosyl hesperidin and residual non-glycosylated hesperidin.
